# Mutation‐related magnetization‐transfer, not axon density, drives white matter differences in premanifest Huntington disease: Evidence from in vivo ultra‐strong gradient MRI

**DOI:** 10.1002/hbm.25859

**Published:** 2022-04-09

**Authors:** Chiara Casella, Maxime Chamberland, Pedro L. Laguna, Greg D. Parker, Anne E. Rosser, Elizabeth Coulthard, Hugh Rickards, Samuel C. Berry, Derek K. Jones, Claudia Metzler‐Baddeley

**Affiliations:** ^1^ Cardiff University Brain Research Imaging Centre (CUBRIC), School of Psychology Cardiff University Cardiff UK; ^2^ Department of Perinatal Imaging and Health, School of Biomedical Engineering & Imaging Sciences King's College London, St Thomas' Hospital London UK; ^3^ Donders Institute for Brain, Cognition and Behavior Radboud University Nijmegen The Netherlands; ^4^ Department of Neurology and Psychological Medicine Hayden Ellis Building Cardiff UK; ^5^ School of Biosciences Cardiff University Cardiff UK; ^6^ Bristol Medical School University of Bristol Bristol UK; ^7^ Birmingham and Solihull Mental Health NHS Foundation Trust Birmingham UK; ^8^ Institute of Clinical Sciences, College of Medical and Dental Sciences University of Birmingham Birmingham UK

**Keywords:** axon, MRI, myelin, premanifest Huntington disease, white matter microstructure

## Abstract

White matter (WM) alterations have been observed in Huntington disease (HD) but their role in the disease‐pathophysiology remains unknown. We assessed WM changes in premanifest HD by exploiting ultra‐strong‐gradient magnetic resonance imaging (MRI). This allowed to separately quantify magnetization transfer ratio (MTR) and hindered and restricted diffusion‐weighted signal fractions, and assess how they drove WM microstructure differences between patients and controls. We used tractometry to investigate region‐specific alterations across callosal segments with well‐characterized early‐ and late‐myelinating axon populations, while brain‐wise differences were explored with tract‐based cluster analysis (TBCA). Behavioral measures were included to explore disease‐associated brain‐function relationships. We detected lower MTR in patients' callosal rostrum (tractometry: *p* = .03; TBCA: *p* = .03), but higher MTR in their splenium (tractometry: *p* = .02). Importantly, patients' mutation‐size and MTR were positively correlated (all *p*‐values < .01), indicating that MTR alterations may directly result from the mutation. Further, MTR was higher in younger, but lower in older patients relative to controls (*p* = .003), suggesting that MTR increases are detrimental later in the disease. Finally, patients showed higher restricted diffusion signal fraction (FR) from the composite hindered and restricted model of diffusion (CHARMED) in the cortico‐spinal tract (*p* = .03), which correlated positively with MTR in the posterior callosum (*p* = .033), potentially reflecting compensatory mechanisms. In summary, this first comprehensive, ultra‐strong gradient MRI study in HD provides novel evidence of mutation‐driven MTR alterations at the premanifest disease stage which may reflect neurodevelopmental changes in iron, myelin, or a combination of these.

## Graphical abstract

This article describes the first application of ultra‐strong‐gradient MRI to separately quantify magnetization transfer ratio (MTR) and hindered and restricted diffusion‐weighted signal fractions, and assess how these measures drive WM microstructure changes in premanifest HD. Findings from this work highlight the fundamental importance of gaining an enhanced understanding of the mechanisms underlying WM abnormalities in HD. Crucially, our results provide novel evidence of mutation‐driven MTR alterations at the premanifest disease stage which may reflect neurodevelopmental changes in iron, myelin, or a combination of these.

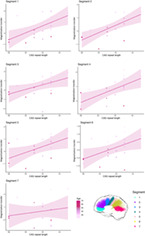

## 1 INTRODUCTION

Huntington disease (HD), a neurodegenerative disorder leading to devastating cognitive, psychiatric, and motor symptoms, cannot currently be cured, and a research priority is to increase understanding of its pathogenesis. Subtle and progressive white matter (WM) alterations have been observed early in HD progression (Casella et al., 2021; Gregory et al., 2019; McColgan et al., 2015; McColgan et al., 2017; McColgan et al., 2018; Paulsen et al., 2008; Scahill et al., 2020), but their etiology and role remain unclear. Therefore, the present study aimed to disentangle the contribution of changes in axon microstructure versus changes in magnetization transfer as a proxy measure of myelin and/or iron, to WM pathology in premanifest HD. Crucially, we exploited the very latest‐in ultra‐strong magnetic field gradient technology (Jones et al., 2018; Setsompop et al., 2013) to achieve high b‐values per unit each time and increased precision in the estimates of hindered and restricted diffusion signal fractions. In turn, this afforded an enhanced differential attenuation of intra‐ and extra‐axonal MRI signals, while maintaining sufficient signal‐to‐noise ratio (SNR), and thus allowed us to better tease apart the contribution of different subcompartments of WM microstructure (Genc et al., 2020; Kleban, Tax, Rudrapatna, Jones, & Bowtell, 2020; Raffelt et al., 2012).

More specifically, we used multimodal quantitative MRI to assess WM microstructure in premanifest patients relative to age‐ and sex‐matched healthy participants, and combined (a) fractional anisotropy (FA), axial diffusivity (AD), and radial diffusivity (RD) from diffusion tensor (DT)‐MRI (Pierpaoli & Basser, 1996), with. (b) The magnetization transfer ratio (MTR) from magnetization transfer imaging (MTI) as a proxy measure of myelin and iron differences. (c) The restricted diffusion signal fraction (FR) from the composite hindered and restricted model of diffusion (CHARMED) (Assaf & Basser, 2005) as a proxy measure of changes in axon density (De Santis, Drakesmith, Bells, Assaf, & Jones, 2014). Alterations in microstructural metrics were assessed using two analytical pipelines: (a) a tractometry approach (Bells, Cercignani, Deoni, et al., 2011; Jones et al., 2006; Jones, Travis, Eden, Pierpaoli, & Basser, 2005), in which the average value of a metric along a specific white matter bundle is derived, to assess tract‐specific changes across the corpus callosum (CC), and (b) a whole‐brain approach (Luque Laguna, 2019) to explore the pattern of abnormalities associated with the premanifest disease stage across all of the brain white matter.

The CC is the brain's largest WM tract and its fibers vary in size and age of myelination, with larger, early myelinating fibers occupying posterior, and smaller, later‐myelinating fibers anterior callosal regions (Aboitiz, Scheibel, Fisher, & Zaidel, 1992). Thus, characterizing WM microstructure across this tract affords insights into the impact of HD on regions with different axonal populations, and may aid in elucidating disease‐related pathological processes in the context of the demyelination hypothesis (Bartzokis et al., 2007). This hypothesis proposes that mutant Huntingtin (mHTT) leads to premature myelin breakdown, and has been given support by several animal studies demonstrating alterations in myelin‐associated biological processes at the cellular and molecular level in the HD brain (Bardile, Garcia‐Miralles, Caron, et al., 2019; Huang et al., 2015; Jin et al., 2015; Radulescu, Garcia‐Miralles, Sidik, et al., 2018; Simmons et al., 2007; Teo et al., 2016; Xiang et al., 2011). For example, electron microscopy investigations have reported thinner myelin sheaths in transgenic BACHD rats and in the HdhQ250 knock‐in mouse model (Jin et al., 2015; Teo et al., 2016). Such alterations in myelin sheaths are paralleled by the reduced expression of myelin‐related genes such as myelin basic protein (MBP) and myelin oligodendrocyte glycoprotein (MOG) in transgenic R6/2 and HdhQ250 knock‐in mice (Blockx, Verhoye, Van Audekerke, et al., 2012; Jin et al., 2015; Xiang et al., 2011). Moreover, these findings are accompanied by evidence of oligodendrocytes alterations provided by both animal and human postmortem studies (Bardile et al., 2019; Ernst et al., 2014; Gómez‐Tortosa, MacDonald, Friend, et al., 2001; Huang et al., 2015; Jin et al., 2015; Myers et al., 1991; Simmons et al., 2007). Specifically, although increased numbers of oligodendrocytes have been observed, evidence suggests that their dysfunctionality may lead to unsuccessful myelination, or that the observed increased levels of oligodendrocytes may be helpful at first but may eventually lead to increased iron toxicity. Both explanations fit within the demyelination hypothesis as they implicate an increasingly unsuccessful compensation for the disease‐related myelin loss. For a critical review of human and animal studies lending support to the demyelination hypothesis (see Casella et al., 2021).

The demyelination hypothesis proposes that myelin impairment begins from early‐myelinating caudate and putamen striatum structures and then spreads in a bilateral and symmetric pattern to other early‐myelinating regions. Thus, in the context of the present study, the demyelination hypothesis would predict more dominant microstructural changes in posterior relative to anterior callosal subregions, as the former myelinate earlier.

Following evidence that WM volume loss in HD extends beyond the CC (Aylward et al., 2011; Beglinger et al., 2007; Ciarmiello et al., 2006; Paulsen et al., 2008; Rosas et al., 2006; Tabrizi et al., 2009; Tabrizi et al., 2011; Tabrizi et al., 2012), and the concept of compensatory networks in response to neurodegeneration (Klöppel et al., 2009), we supplemented the tractometry analysis with a novel exploratory, whole‐brain analysis, called tract‐based cluster analysis (TBCA) (Luque Laguna, 2019) to assess brain‐wise group microstructural differences. TBCA uses the rich anatomical information from whole‐brain tractography reconstructions to inform the cluster‐level inference analysis of voxel‐based images, and provides the anatomical specificity required to disentangle distinct clusters belonging to different anatomical tracts (Luque Laguna, 2019).

Finally, the evidence of cognitive and behavioral impairments in premanifest patients (Landwehrmeyer et al., 2017; Paulsen et al., 2008; Tabrizi et al., 2012) across attention, working memory, processing speed, psychomotor functions, episodic memory, emotion processing, sensory‐perceptual functions, and executive functions (Paulsen et al., 2008; Paulsen et al., 2014; Paulsen et al., 2014; Paulsen, Miller, Hayes, & Shaw, 2017; Pirogovsky et al., 2009; Stout et al., 2011; Stout et al., 2012), and their significant impact on everyday functional decline (Hamilton et al., 2003; Nehl et al., 2004; Williams et al., 2010), stress the importance of understanding how these symptoms may relate to pathological neural changes, such as alterations in WM microstructure. For this purpose, we derived a composite cognitive score using principal component analysis (PCA) to capture variability in patients' cognitive performance and then used it for the analysis of correlations between differences in cognition and WM microstructure.

## 2 MATERIALS AND METHODS

### 2.1 Participants

Twenty‐five individuals with premanifest HD and 25 age‐ and sex‐matched healthy controls were recruited, with ethical approval from the local National Health Service (NHS) Research Ethics Committee (Wales REC 5 18/WA/0172) and by the Cardiff University School of Psychology Ethics Committee. All participants provided written informed consent prior to taking part in the study.

Patients were recruited from the Cardiff HD Research and Management clinic, Bristol Brain Centre at Southmead Hospital, and the HD clinic at the Birmingham and Solihull NHS Trust. Healthy controls were recruited from Cardiff University and the School of Psychology community panel. Participants were recruited if eligible for MRI scanning. Control participants were excluded if they had a history of neurological or psychiatric conditions, and patients if they had a history of any other neurological condition.

Twenty‐two of the HD patients had pen‐and‐paper cognitive task data available from their most recent participation in the ENROLL‐HD study (NCT01574053, https://enroll-hd.org). The progression of symptoms in ENROLL‐HD participants is monitored longitudinally, and one of the optional components within the study is the giving of permission by participants for their coded data to be accessed by researchers in the field. As such, a full clinical dataset including full medical and medication history is available for each research participant and some of these data were used in this study.

One control subject was excluded from the tractometry analysis because of poor callosal segmentation. Therefore, data from 25 patients and 24 healthy controls were used for callosal tractometry analysis. As the callosal segmentation did not impact TBCA, a sample of 25 patients and 25 controls was analyzed. Table 1 provides a summary of participants' demographic and clinical background information. Performance in the montreal cognitive assessment (MoCA) (Nasreddine et al., 2005) and in the test of premorbid functioning—UK Version (TOPF‐UK) (Wechsler, 2011) is reported for patients and controls. The unified Huntington disease rating scale (UHDRS), total motor score (TMS), total functional capacity (TFC), diagnostic confidence level (DCL), and CAG repeat size obtained from the ENROLL‐HD database are also reported for patients.

**TABLE 1:** Summary of participants' demographic and clinical background information

	HD patients	Controls	*p*‐Value
Gender: male/female (%)	15(60)/10(40)	14(56)/11(44)	*p* > .05
Mean age (years) (SD, range)	42.04 (12.7, 21–70)	43.19 (12.6, 27–71)	*p* > .05
Mean TOPFUK IQ (SD, range)	116.16 (10.2, 98–137.4)	124.96 (6.9, 109–135.4)	***p* = .003**
Mean MoCA score (SD, range)	27.92 (2.1, 24–30)	28.2 (1.8, 26–30)	*p* > .05
Mean CAG (SD, range)	41.4 (2.1, 37–45)	‐	‐
Mean DBS (SD, range)	235.94 (84.5, 61.5–450)	‐	‐
Mean TFC (SD, range)	12.863 (0.4, 12–13)	‐	‐
Mean TMS (SD, range)	3.3 (4.8, 0–18)	‐	‐
Mean DCL (SD, range)	0.91 (1.3, 0–3)	‐	‐

*Note*: TOPFUK FSIQ = verbal IQ estimate based on the Test of Premorbid Functioning, UK version. There was a significant difference between patients and controls in TOPFUK FSIQ, with patients presenting significantly lower premorbid IQ. MoCA = Montreal Cognitive Assessment out of 30 (the higher the score the better the performance). MoCA scores for patients and controls ranged between 23 and 30. A score of 26 or over is generally considered to be normal, while an average score of 22.1 has been reported in people with mild cognitive impairment (Nasreddine, Phillips, Bédirian, et al., 2005). There was no significant difference in this test between the two groups. Two individuals with CAG repeats of 38 were included in the current study. Although these individuals can be considered “affected,” they may have a lower risk of becoming symptomatic within their life span; DBS = Disease Burden Score, calculated as follows: DBS = age × (CAG‐35.5); TMS = Total Motor Score out of 124 from “UHDRS Motor Diagnostic Confidence (Motor)—the higher the score, the more impaired the performance. Based on TMS scores, all patients were at the premanifest disease stage. DCL = Diagnostic Confidence Level (normal/no abnormalities = 0, nonspecific motor abnormalities = 1, motor abnormalities that may be signs of HD = 2, motor abnormalities that are likely signs of HD = 3, motor abnormalities that are unequivocal signs of HD = 4). Only participants with diagnostic confidence level ratings < 4 were included in the current report. However, based on DCL scores, some of the patients (*n* = 4) presented with some motor abnormalities. Values below .05 are considered significant.

## 3 DATA ACQUISITION

### 3.1 Assessment of disease‐related brain‐function relationships

A composite cognitive score was computed by combining cognitive data available for patients on the ENROLL‐HD database (providing these had been obtained within a 3‐month time window from their participation in the present study), with data acquired during the study. This was done in order to reduce patient burden associated with study participation. Table 2 provides details on the administered tests, the cognitive domains they assess, and the outcome variables measured.

**TABLE 2:** Cognitive outcome variables employed to create a composite cognitive score to assess disease‐related brain‐function relationships

Task	Computerized/paper & pencil	Description	Outcome variable	Cognitive domain assessed
N‐back (Kirchner, 1958)	Computerized	Participants were presented with a series of letters, 3 s apart, and asked to judge whether the current letter matched the previous letter (one‐back condition) or the letter presented two letters back (two‐back condition). The one‐back and two‐back conditions were presented separately in 20 randomly ordered trials. Participants made responses manually by pressing on the letter “A” on the keyboard. No responses were required for nontargets.	Percentage of correct responses in the one‐back and two‐back condition	Encoding, temporary storage and updating of stored information with new upcoming information, inhibition of irrelevant items
Digit span test from the WAIS‐R (Wechsler, 1997)	Computerized	Participants were presented with a series of numbers that appeared on the screen one after another. They were required to recall the sequence of numbers by entering them on the keyboard. If the participant could successfully reproduce the series of numbers, they were then presented with a longer series of numbers. Participants continued to receive longer series of numbers until they could no longer repeat them back correctly. The starting list length was 3, and the longest list length possible was 10. The discontinuation criterion was two wrong responses.	Maximum span of digits recalled	Verbal working memory capacity
Visual patterns test (Della Sala, Gray, & Baddeley, 1997)	Paper and pencil	Participants were shown a checkerboard‐like grid, with the squares in the grid each randomly colored. This pattern was displayed for 3 s and is then removed. Subjects were then shown a blank grid and were asked to reproduce each grid. The number of items was sequentially increased. Participants were given unlimited time to reproduce the shapes being viewed.	Maximum grid size recalled correctly	Spatial working memory capacity
Speeded finger tapping task (Reitan & Davison, 1974)	Computerized	Participants were instructed to form a fist shape with their dominant hand, with their fingernails touching down in front of the keyboard space bar. They were then instructed to extend their index finger in order to contact the “space” bar on the keyboard, and to move only their index finger to tap the space bar as quickly as possible.	Mean number of taps over three trials	Motor speed
Stroop interference, word Reading and color naming (Kieburtz, Penney, Corno, et al., 2001; Movement Disorders, 1996; Siesling, Van Vugt, Zwinderman, Kieburtz, & Roos, 1998)	Paper and pencil	For the Stroop Reading and color naming, participants had to name colors (e.g., red, green, blue) and read the words for colors in black ink. For the Stroop interference, participants had to read words of colors (e.g., red, green blue) where the word color was written in a different color ink (Stroop interference).	Number of correct responses	Ability to inhibit cognitive interference, selective attention capacity and skills, processing speed, motor control
Phonetic and category verbal fluency (Kieburtz et al., 2001; Movement Disorders, 1996; Siesling et al., 1998)	Paper and pencil	In the phonetic verbal fluency task participants had to spontaneously produce words orally within a fixed time span (60 s), beginning with a certain letter. In the category verbal fluency, words had to be produced according to semantic constraints (e.g., animals, fruits, vegetables).	Number of correctly generated words within 60 s	Working memory, cognitive inhibition, switching ability and language ability including lexical knowledge and lexical retrieval ability
Trail making (part A & part B) (Kieburtz et al., 2001; Movement Disorders, 1996; Siesling et al., 1998)	Paper and pencil	In part A, participants were asked to connect 25 randomly arrayed dots in numerical order, whereas in part B they were asked to connect dots alternating between numbers and letters in alphabetical order.	Time needed to complete the task	Visual attention, task switching, speed of processing, mental flexibility
Symbol digit modality (Kieburtz et al., 2001; Movement Disorders, 1996; Siesling et al., 1998)	Paper and pencil	Using a reference key, participant had 90 s to pair specific numbers with given geometric figures.	Number of correct responses achieved in 90 s	Attention, perceptual speed, motor speed, and visual scanning

*Note*: Tasks descriptions are provided, outcome variables and cognitive domains assessed are summarized.

Briefly, data from the ENROLL‐HD database concerned performance in the phonetic verbal fluency test, the categorical verbal fluency test, the symbol digit modality test, the stroop color reading and word reading test, the stroop interference test and the trail making test (Kieburtz et al., 2001; *Movement Disorders*, 1996; Siesling et al., 1998)—see http://www.enroll-hd.org for the detailed study protocol.

On the other hand, performance in the N‐back task (Kirchner, 1958), the forward digit span test adapted from the wechsler adult intelligence scale‐revised (WAIS‐R) (Wechsler, 1997), the visual patterns test, (Della Sala et al., 1997) and the speeded finger tapping task (Reitan & Davison, 1974) was assessed as part of the present study. Cognitive testing was performed prior to MRI scanning and lasted approximately 60 min. Tasks were administered either as paper and pencil tests or by using a computerized version provided by the psychology experiment building language (PEBL) test battery (Mueller & Piper, 2014).

As each task yields several outcome variables, the following strategy was employed: (a) for standardized clinical tests, metrics known to have the best sensitivity and measurement characteristic were selected, for example, correctly generated responses instead of error scores (Metzler‐Baddeley et al., 2014); (b) for tests with multiple conceptually distinct outcome measures, variables that represented each component were included, for example, for the N‐back task, the number of correct responses from the 1‐back and the 2‐back condition; and (c) where necessary, variables were excluded from the assessment, for example, when these presented lots of missing cases. This approach led to 13 cognitive outcome measures (Table 2).

### 3.2 MRI data acquisition

MRI data were acquired on a 3 Tesla Siemens Connectom system with ultra‐strong (300 mT/m) gradients. Each MRI session lasted 1 hr, and comprised: a T_1_‐weighted MPRAGE; a multishell dMRI acquisition (δ/Δ: 7/24 ms; b‐values: 0 [14 volumes, interleaved], 500 [30 directions], 1,200 [30 directions], 2,400 [60 directions], 4,000 [60 directions], and 6,000 [60 directions] s/mm^2^ (Tournier, Calamante, & Connelly, 2013). Data were acquired in an anterior–posterior phase‐encoding direction, with one additional posterior‐to‐anterior volume); and a magnetization transfer acquisition (turbo factor: 4; radial reordering; nonselective excitation; MT contrast was achieved by the application of a 15.36 ms radio‐frequency saturation pulse, with an equivalent flip angle of 333° applied at a frequency of 1.2 kHz below the water resonance. Two identical sets of images with different contrasts [one acquired with and one acquired without MT saturation pulses] were obtained). Table 3 provides more details on the acquisition parameters.

**TABLE 3:** Scan parameters

	T_1_‐w	DTI	CHARMED	MT
Pulse sequence	MPRAGE	SE\EPI	SE\EPI	Turbo FLASH
Matrix size	256 × 256	495 × 495	495 × 495	128 × 128 × 104
FoV (mm)	256	990	990	220 × 220 × 179
Slice thickness (mm)	1	2	2	1.72
TE,TR (ms)	2, 2,300	59, 3,000	59, 3,000	2.1, 60
Off‐resonance pulses (Hz/°)	‐	‐	‐	1200/333
Flip angles (°)	9	90	90	5

*Note*: All sequences were acquired at 3 T with ultra‐strong gradients. For each of the sequences, the main acquisition parameters are provided.

Abbreviations: EPI, echo‐planar imaging; FoV, field of view; MPRAGE, Magnetization prepared—rapid gradient echo; MT, magnetization transfer; SE, spin‐echo; T_1_‐w, T_1_‐weighted; TE, echo time; TR, repetition time.

## 4 IMAGE PROCESSING

All images were skull‐stripped in native space using FSL BET (Smith et al., 2004).

### 4.1 Diffusion data: FA, RD, AD, MD, and FR maps

Preprocessing of diffusion data was carried out using FMRIB Sofware Library (FSL) (Smith et al., 2004), MRtrix3 (Tournier et al., 2019), and advanced normalization tools (ANTs) (Avants et al., 2011). These steps included: denoising (Veraart, Fieremans, & Novikov, 2016), slice‐wise outlier detection (SOLID) (Sairanen, Leemans, & Tax, 2018), and correction for drift (Vos et al., 2017); motion, eddy, and susceptibility‐induced distortions (Andersson, Skare, & Ashburner, 2003; Andersson & Sotiropoulos, 2016); Gibbs ringing (Kellner, Dhital, Kiselev, & Reisert, 2016); bias field (Tustison et al., 2010); and gradient nonlinearities (Glasser et al., 2013; Rudrapatna, Parker, Roberts, & Jones, 2021).

Diffusion tensors were estimated using linearly weighted least squares regression (for *b* < 1,200 s/mm^2^ data) providing the following quantitative scalar measures: FA, AD, and RD. The diffusion tensor was fitted to data between *b* = 500 s/mm^2^ and *b* = 1,200 s/mm^2^ in order to reduce cerebrospinal fluid‐based partial volume artifacts in the DTI metrics. The CHARMED data were corrected for motion and distortion artifacts (Ben‐Amitay, Jones, & Assaf, 2012), before computing FR maps (Assaf & Basser, 2005) using in‐house software coded in MATLAB (The MathWorks, Natick, MA).

### 4.2 Magnetization transfer: MTR Maps

MT‐ and non‐MT‐weighted images were corrected for Gibbs ringing (Kellner et al., 2016). ANTS (Avants et al., 2011) was first used to nonlinearly register the MPRAGE images to the *b* = 0 s/mm^2^ images. Then, MT‐ and non‐MT weighted images were linearly warped to the registered MPRAGE images using an affine (12° of freedom) technique based on mutual information, with the FMRIB's linear image registration tool (FLIRT) (Jenkinson & Smith, 2001). All registrations were visually inspected for accuracy. Finally, MTR maps were calculated according to: MTR = ([S^0^ − S^MT^]/S^0^) × 100, whereby S^0^ represents the signal without the off‐resonance pulse and S^MT^ represents the signal with the off‐resonance pulse.

### 4.3 Tractography of the CC

**FIGURE 1:**
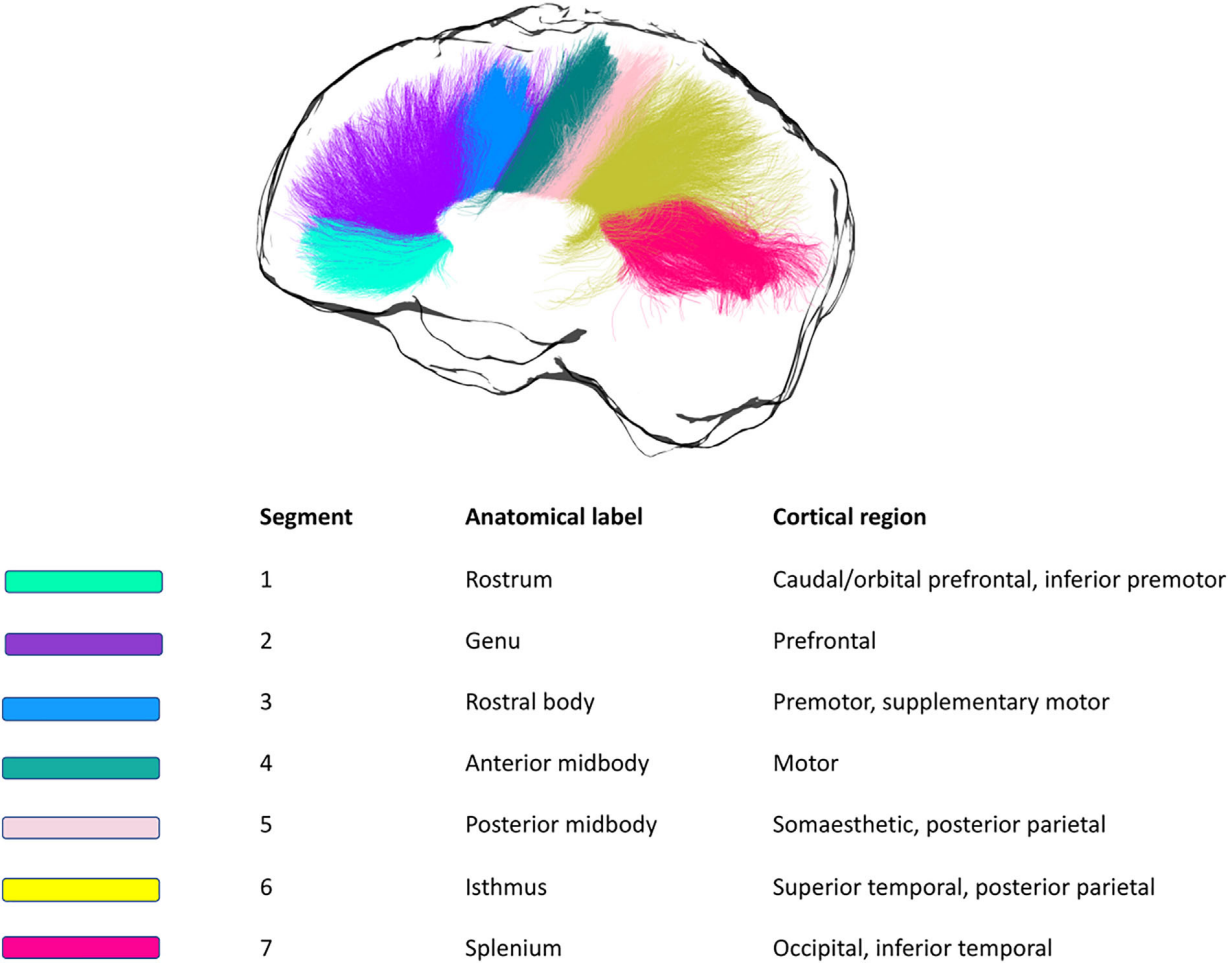
Callosal segmentation. For each segment, the corresponding anatomical label is reported, together with the cortical area it connects to

Automated WM tract segmentation of the CC was performed using TractSeg (Wasserthal, Neher, & Maier‐Hein, 2018) and multishell‐constrained spherical deconvolution (MSMT‐CSD) (Jeurissen, Tournier, Dhollander, Connelly, & Sijbers, 2014). Specifically, seven portions of the CC were delineated (1 = rostrum, 2 = genu, 3 = rostral body, 4 = anterior midbody, 5 = posterior midbody, 6 = isthmus, 7 = splenium) (Figure 1). For each segment, 2,000 streamlines were generated.

## 5 STATISTICAL ANALYSIS

Analyses were performed in RStudio (Team Rs, 2015), MATLAB (The MathWorks), SPSS (Armonk, 2011), the PROCESS computational tool for mediation analysis (Hayes, 2017), FSL (Smith et al., 2004), and the statistical nonparametric mapping (SnPM) software (Nichols & Holmes, 2014). Outliers were first identified by examining box‐and‐whisker plots for each dependent variable, for controls and patients separately. Outliers that were ±3*SD*s from the mean were removed.

### 5.1 Assessment of disease‐related brain‐function relationships

PCA of the cognitive data was performed on the slopes of the patient data to best capture heterogeneity within this population. Only the first principal component (PC) was extracted, to increase experimental power and reduce the number of multiple comparisons (Steventon, 2014).

First, the Bartlett's test of sphericity and the Kaiser‐Meyer‐Olkin (KMO) test were used to confirm that the data were suited for PCA (KMO = 0.54, *χ* ^2^(78) = 156.5, *p* < .001). The PCA was run using centered, standardized versions of the patients' cognitive outcome scores. Orthogonal varimax rotation was used to maximize the factor loadings. Regression values from each component were used as composite cognitive scores for each patient.

### 5.2 Tractometry of the CC

Microstructure differences were assessed in the seven callosal segments. By taking each quantitative metric map, samples of each metric were obtained at each vertex of the reconstructed segments, and segment‐specific medians were derived for FA, AD, RD, FR, and MTR in MRtrix3 (Tournier et al., 2019). Next, the overall mean was calculated, so that each dataset comprised *m* = 5 MRI‐derived measures, mapped along *s* = 7 callosal segments.

### 5.3 Reduction of MRI data dimensionality with PCA

PCA was also employed to reduce the complexity of the callosal microstructure data (Geeraert, Chamberland, Lebel, & Lebel, 2020). Centered, standardized versions of MRI measures on both groups combined were used (Phillips et al., 2013). Specifically, the PCA was calculated for FA, FR, RD, AD and MTR, after checking that the data was suited for this analysis (KMO = 0.65, *χ* ^2^(6) = 1077.231, *p* < .001). PCA was applied to the concatenated set of segments across subjects (Chamberland et al., 2019; Wickham, 2014). The number of principal components was extracted based on: (a) their interpretability (Metzler‐Baddeley et al., 2017); (b) the Kaiser criterion of including all components with an eigenvalue greater than 1. Regression values from each component for each participant were used in the following analyses.

### 5.4 Investigation of group differences in callosal microstructure

To assess group differences in callosal microstructure, analyses of covariance (ANCOVAs) were run on the extracted regression values from each component for each participant. Group and segment were used as independent variables because of a particular interest in understanding the interaction between group effects on different callosal segments. The correlation of microstructure outcome measures across patients and controls, with age, ICV, and TOPF‐UK FSIQ was tested to decide if these variables should be included as covariates in the analysis. Pearson's correlation coefficients greater than 0.3 were treated as indicative of a moderate relationship. For every ANCOVA, analysis assumptions were first tested.

### 5.5 Assessment of disease‐related brain‐function relationships

Spearman correlations were run in the patient group for:

1. The extracted regression values from each significant component for each participant, and their respective composite cognitive scores;
2. The extracted regression values from each significant component for each participant, and their respective CAG repeat length;
3. The extracted regression values from each significant component for each participant, and their respective disease burden score (DBS), calculated as follows: DBS = age × (CAG‐35.5).

Within each group of correlations, multiple comparison correction was carried out with Bonferroni with a family‐wise alpha level of 5% (two‐tailed). Whenever a significant association was detected, this was further explored with partial correlations, partialling out ICV and DBS. The latter was done to assess associations independently of disease progression.

### 5.6 TBCA assessment of brain‐wise group differences in WM microstructure

TBCA (Luque Laguna, 2019) was applied to assess group differences in FA, RD, AD, FR, and MTR. This method is based on the novel concept of a “hypervoxel,” which extends standard 3D voxels with extra dimensions to encode geometrical and topological information about the streamlines that intersect each voxel.

**FIGURE 2:**
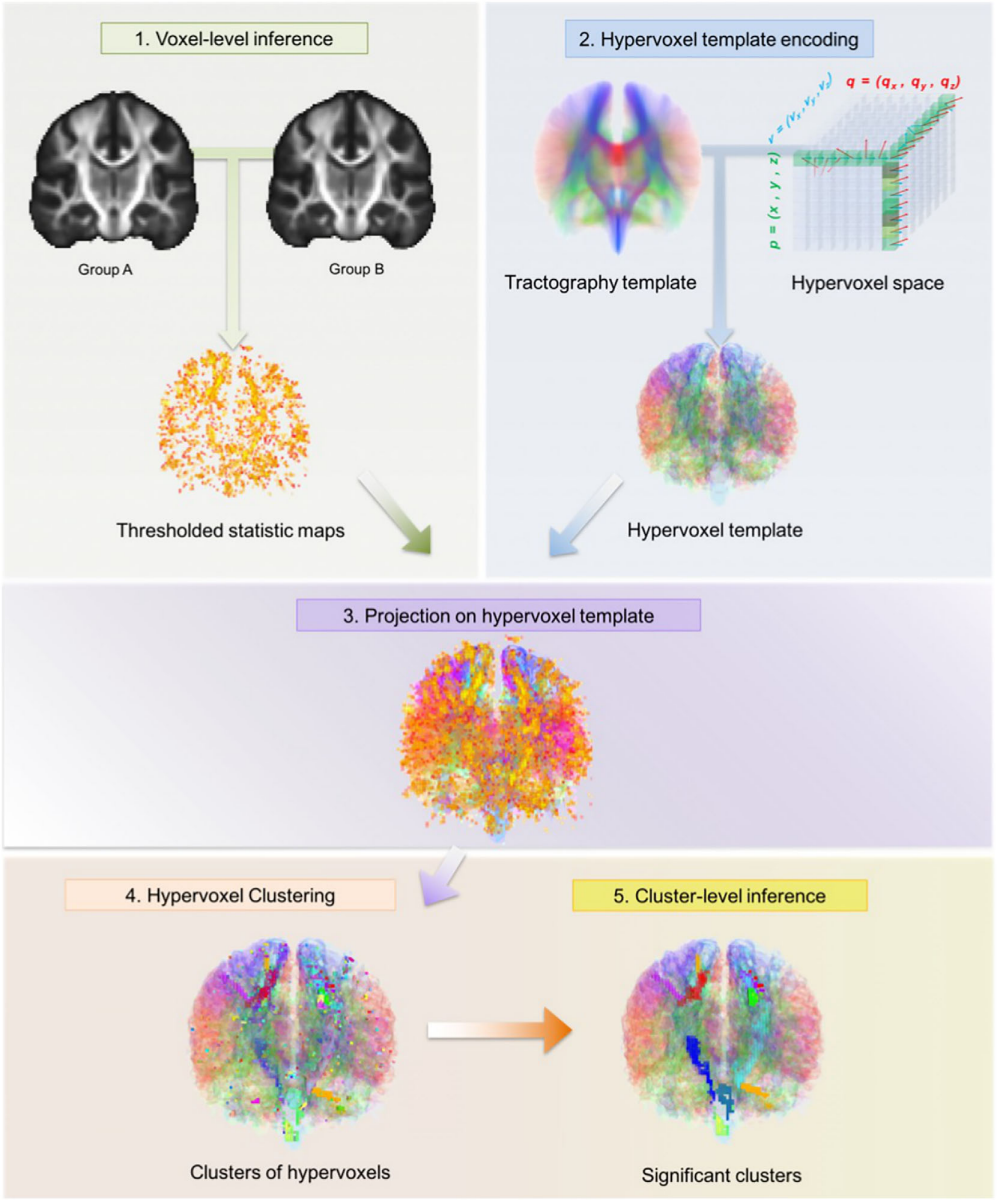
The TBCA analysis pipeline. After all images have been normalized to a common anatomical space, statistics maps are produced based on the voxel‐level analysis of the data; this is done by using a nonparametric approach based on a permutation test strategy (Winkler et al., 2014). The statistic maps are thresholded by a value of *p* = .01. Next, the significant voxel level statistic results are projected on a hypervoxel template. Finally, significant clusters of hypervoxels are identified. Figure from Luque Laguna (2019)

All images were first nonlinearly normalized to the FMRIB58_FA template (1 × 1 × 1 mm isotropic) using the tbss_2_reg script (Smith et al., 2006). Next, statistical maps were produced based on the voxel‐level analysis of the data by using a nonparametric approach based on a permutation test strategy (Winkler, Ridgway, Webster, Smith, & Nichols, 2014). The statistical maps were then thresholded at *p* = .01, and the suprathreshold voxel‐level statistic results were projected onto an hypervoxel template built on whole‐brain tractography data from 20 healthy subjects. Two hypervoxels were defined as belonging to the same cluster if they were either adjacent or connected within the hypervoxel template (i.e., if they shared a common streamline) (Luque Laguna, 2019). Finally, the mass of each cluster (Bullmore et al., 1999) was computed and their corresponding statistical significance calculated based on the same permutation tests used for the voxel‐level inference. Explanatory variables (EVs) in the permutation tests included age and gender and the effect of group was explored while regressing the other EVs. Clusters with a family‐wise error (FWE)‐corrected (Nichols & Holmes, 2014) *p*‐value below .05 were considered statistically significant. A schematic representation of the TBCA pipeline can be found in Figure 2.

Whenever significant clusters were detected for a specific metric, these were extracted, summed, and binarized to form an ROI mask. The mask was then projected onto each map in MNI space. The mean value for that metric was calculated in the ROI with FSL (Smith et al., 2004), and used to run Spearman correlations between the WM metrics showing significant clusters. Multiple comparison correction was carried out with the Bonferroni correction with a family‐wise alpha level of 5% (two‐tailed).

## 6 RESULTS

### 6.1 Composite cognitive score in the patient sample

**FIGURE 3:**
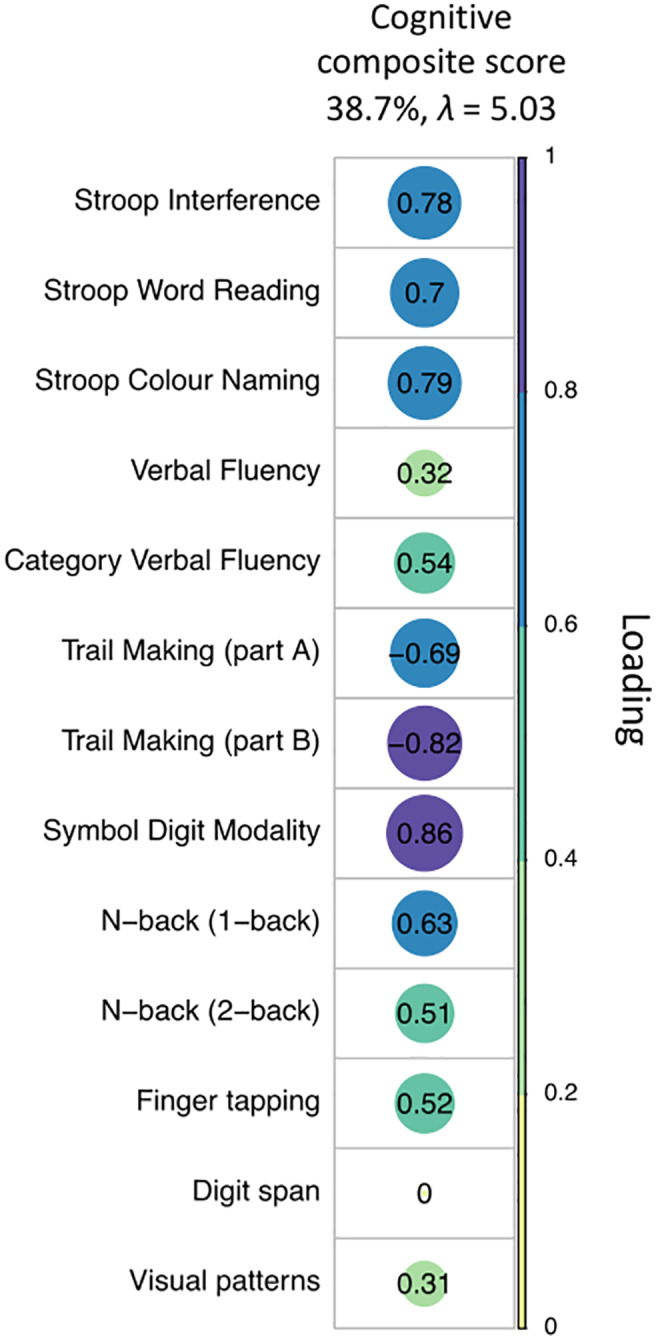
PCA of the cognitive data with varimax rotation. Plot summarizing how each variable is accounted for in the extracted PC. The absolute correlation coefficient is plotted. Color intensity and the size of the circles are proportional to the loading. This PC accounted for 38.7% of the total variance and included measures from all test domains, except for the digit span. Four patients were excluded from the PCA because of missing data. The final sample size for the PCA was *n* = 21 patients

As shown in Figure 3, the first principal component (PC) accounted for 38.7% of the total variance in the cognitive data. Component loadings of ≥0.5 were considered as significant (Bourbon‐Teles et al., 2019). Thus, this component reflected general executive functioning with loadings on distractor suppression (Stroop task), attention switching (Trail Making), updating (N‐back), category fluency, and motor speed.

### 6.2 Reduction of MRI data dimensionality with PCA

**FIGURE 4:**
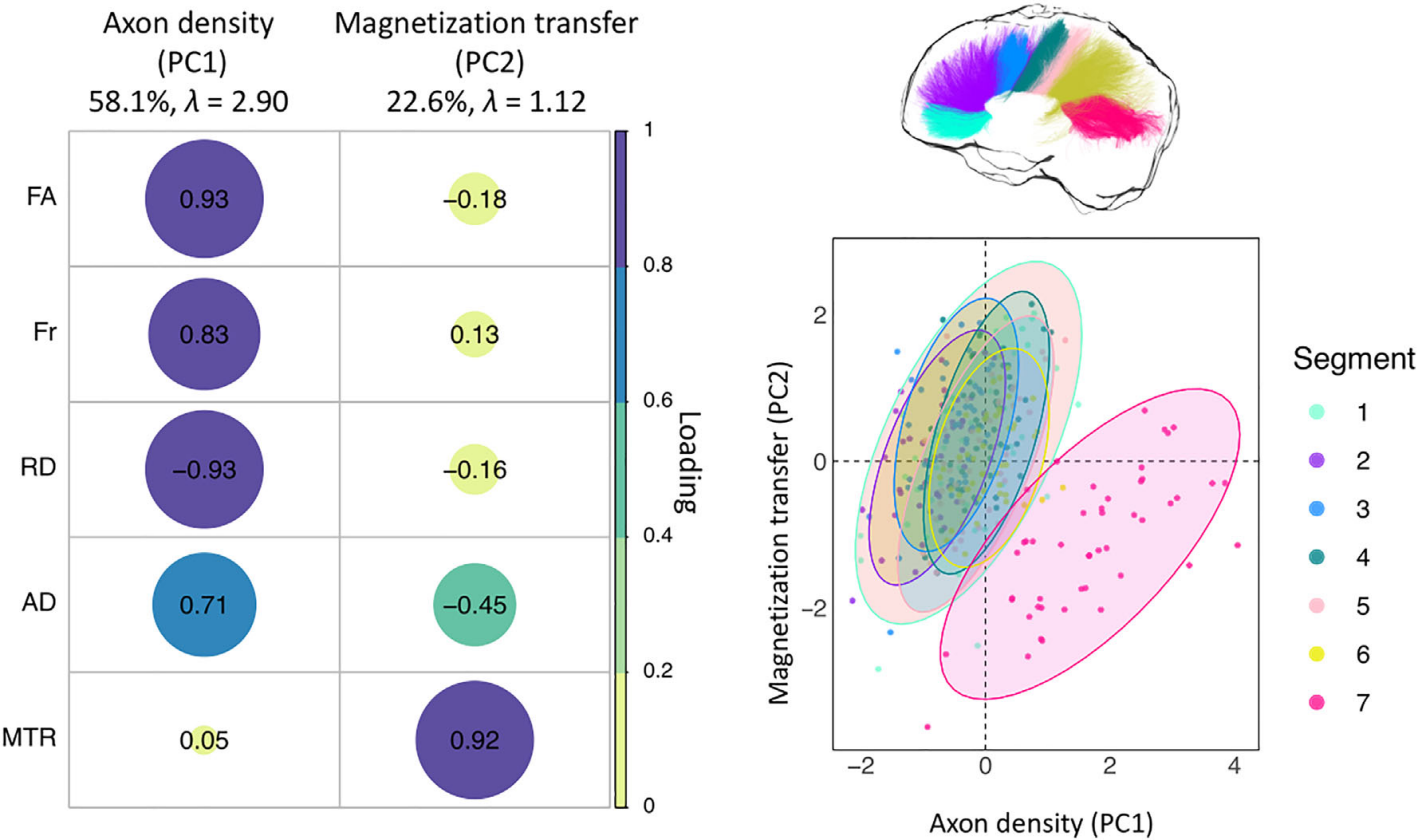
PCA of the microstructure metrics with varimax rotation. Left: Plot summarizing how each variable is accounted for in every principal component. The absolute correlation coefficient is plotted. Color intensity and the size of the circles are proportional to the loading. The final sample size for the PCA was *n* = 25 for the HD group and *n* = 24 for the control group. Right: Segment clustering based on PC1 and PC2. The horizontal axis shows increasing restriction or hindrance perpendicular to the main axis of the bundles. The vertical axis represents an increase in MTR. Each point represents one subject. Concentration ellipsoids cover 95% confidence around the mean. Segment 7 appears to encompass most of the data variability

Over 80% of the variability in the microstructure data was accounted for by the first two principal components (PC1, 58.1%, *λ* = 2.90; PC2, 22.6%, *λ* = 1.13). As shown in Figure 4, the first PC loaded positively on FA, FR, and AD, and negatively on RD, measuring restriction or hindrance perpendicular to the main axis of the bundle, and was therefore summarized as “axon density” component. The second component loaded mostly on MTR and was thus summarized as “magnetization transfer” component.

### 6.3 Premanifest patients present alterations in callosal MTR but not axon density

#### 6.3.1 Assessment of group differences in axon density

Age was negatively associated with axon density scores (*r* = −0.301, *p* < .001), and included in the final model assessing the effect of group and segment on axon density scores, with age as covariate.

The effect of group was not significant (*F*(1, 312) = 1.677, *p* = .196), however, a main effect of segment was detected (*F*(6, 312) = 84.671, *p* < .001) (Figure 4), together with a main effect of age (*F*(1, 312) = 34.116, *p* < .001) (Figure 4). The Group × Segment interaction was not significant (*F*(6, 312) = 0.531, *p* = .784). Overall, age was negatively associated with scores on this component; additionally, microstructure in the posterior segments of the CC was associated with higher axon density scores, compared to anterior ones (adjusted means: CC1 = −0.270; CC2 = −0.822; CC3 = −0.546; CC4 = −0.001; CC5 = −0.144; CC6 = 0.083; CC7 = 1.753).

#### 6.3.2 Assessment of group differences in the magnetization transfer component

Age and ICV were correlated with scores on the magnetization transfer component (age: *r* = −0.301, *p* < .001; ICV: *r* = −0.332, *p* < .001), thus the final model assessed the main effects of group and segment, and age‐by‐group and a group‐by‐segment interactions, with age as covariate.

There were no main effects of group (*F*(1, 312) = 2.353, *p* = .126) or ICV (*F*(1, 312) = 1.875, *p* = .172). However, significant main effects of age (*F*(1,312) = 45.07, *p* < .001) and segment (*F*(1, 312) = 19.899, *p* < .001) were detected. Overall, scores on this component were lower in segment 7 of the CC and in older participants (Figure 4).

Crucially, a significant interaction was detected between segment and group (*F*(6, 312) = 2.238, *p* = .040), indicating that the effect of group was different for different callosal segments. Therefore, slopes of the effect of group on PC2 for each segment, while controlling for the effect of age, were investigated with a simple moderation analysis using the PROCESS toolbox for SPSS (Hayes, 2017), to better understand this interaction.

**FIGURE 5:**
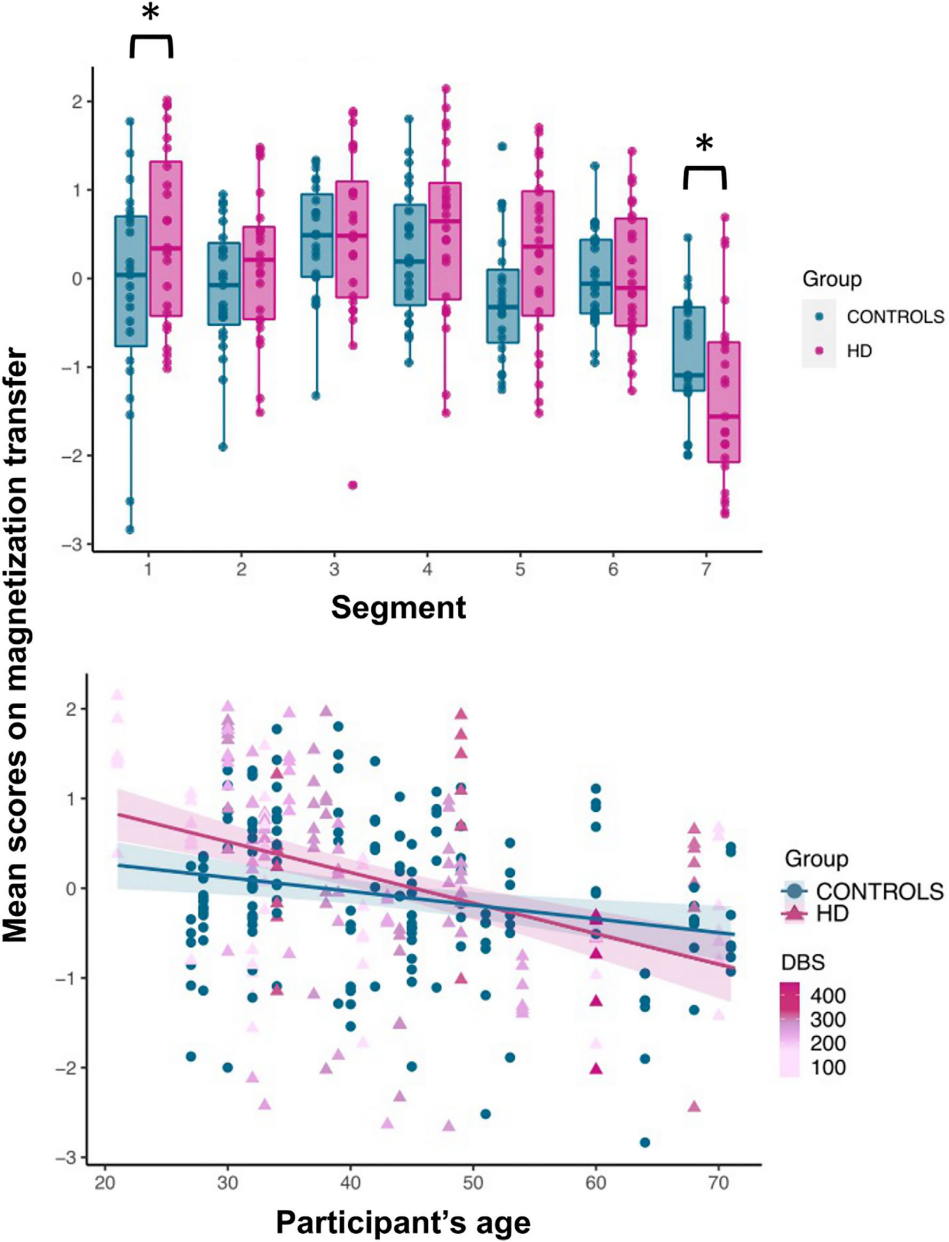
Callosal magnetization transfer: patient‐control differences across callosal segments (top), and relationship between age and inter‐individual variability in the magnetization transfer component (bottom). A group‐by‐segment interaction effect (*p* = .04) was observed for callosal magnetization transfer, indicating that the effect of group was different for different callosal segments. Patients presented significantly higher magnetization transfer compared to controls in segment 1 (*p* = .016), and significantly lower in segment 7 (*p* = .034). Overall, scores on the magnetization transfer component for the patient group were higher than controls in the more anterior portions of the CC but lower in posterior portions. Additionally, a significant interaction effect between group and age indicated that, while older HD patients presented significantly lower magnetization transfer than age‐matched controls, the opposite was true for younger HD patients. *p < .05, **p < .01, ***p < .001, Bonferroni‐corrected

This analysis revealed that patients presented significantly higher scores on the magnetization transfer component compared to controls in segment 1 (*p* = .016), and significantly lower scores in segment 7 (*p* = .0343). Overall, scores on this component for the patient group were higher than controls in the more anterior portions of the CC but lower in the posterior portions (segment 1: *β* = 0.56, *t* = 2.41, *p* = .016; segment 2: *β* = 0.25, *t* = 1.08, *p* = .27; segment 3: *β* = 0.014, *t* = 0.06, *p* = .95; segment 4: *β* = 0.2098, *t* = 0.90, *p* = .36; segment 5: *β* = .44, *t* = 1.89, *p* = .058; segment 6: *β* = −0.028, *t* = −0.12, *p* = .899; segment 7: *β* = −0.5, *t* = −2.12, *p* = .034) (Figure 5).

As a post hoc, exploratory analysis, the impact of partial volume artifacts on magnetization transfer differences between patients and controls was assessed. The fractional volume of free water in each voxel was estimated from the diffusion data to produce a free‐water signal fraction (FWF) map. The overall mean FWF was then calculated, as described above for the other metrics assessed. Finally, an ANCOVA was run to assess group differences in magnetization transfer across the different segments, controlling for FWF. Specifically, the main effects of group and segment and their interaction effect were examined, with age, ICV and FWF as covariates. Age‐by‐group and group‐by‐FWF interactions were included in the model because of violation of the homogeneity of regression slopes assumption.

Consistent with the main analysis, a significant main effect of age (*F*(1, 300) = 56.08, *p* < .001) and segment (*F*(1, 300) = 22.89, *p* < .001) and a significant interaction effect between segment and group (*F*(1,300) = 3.2, *p* = .005) were detected. The interaction between group and age (*F*(1, 300) = 8.736, *p* = .003) was now significant, indicating that while scores on this component are lower than age‐matched controls in older patients, the opposite was true for younger patients. Finally, a significant main effect of group (*F*(1, 300) = 13.042, *p* < .001), and FWF (*F*(1, 300) = 13.32, *p* < .001), and a significant interaction effect between group and FWF (*F*(1, 300) = 19.262, *p* < .001), were detected.

### 6.4 Magnetization transfer is associated with CAG repeat length but not with cognitive performance or disease burden

**FIGURE 6:**
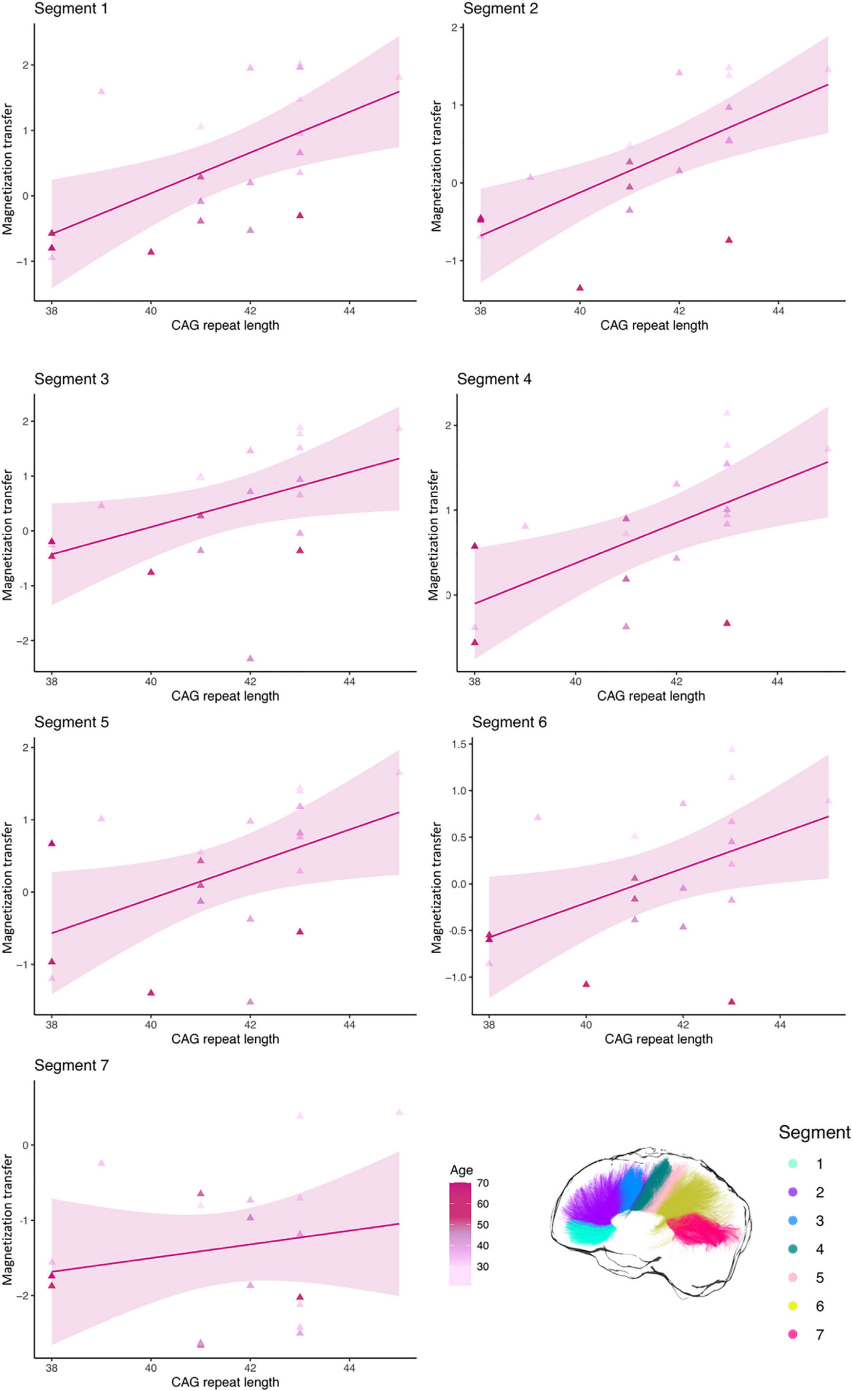
Relationship between magnetization transfer in each callosal segment and CAG repeat length in patients

Spearman correlation coefficients and associated *p*‐values for the correlations of magnetization transfer with composite cognitive scores, CAG repeat length and DBS are reported in Table 4. Trends for positive associations were detected between composite cognitive scores and magnetization transfer in all segments, except for segment 7. However, these associations were no longer significant after multiple comparison correction. Magnetization transfer was positively correlated with CAG repeat length in segment 1 (*r* = 0.641, *p* = .002), segment 2 (*r* = 0.717, *p* = .001), segment 3 (*r* = 0.549, *p* = .012), segment 4 (*r* = 0.549, *p* = .012), segment 5 (*r* = 0.525, *p* = .018), and segment 6 (*r* = 0.513, *p* = .021). After Bonferroni correction the relationship remained significant in segments 1 (*p* = .014), 2 (*p* = .007), and 4 (*p* = .007) (Figure 6). Partial correlations were carried out to explore the relationships between magnetization transfer and CAG repeat length independently of ICV and disease burden. Even stronger positive associations were now detected; interestingly, the association was now significant also in segment 7, before correction (segment 1: *r* = 0.763, *p* = .001, corrected *p* = .007; segment 2: *r* = 0.879, *p* < .001, corrected *p* < .001; segment 3: *r* = 0.841, *p* < .001, corrected *p* < .001; segment 4: *r* = 0.83, *p* < .001, corrected *p* < .001; segment 5: *r* = 0.745, *p* = .001, corrected *p* = .007; segment 6: *r* = 0.864, *p* < .001, corrected *p* < .001; segment 7: *r* = 0.5, *p* = .048, corrected *p* = 0.336) (Figure 5). No significant associations were detected between magnetization transfer scores in each of the seven callosal segments and DBS.

**TABLE 4:** Correlations of magnetization transfer scores with cognitive component scores, CAG repeat‐length, and DBS

Magnetization transfer	Composite cognitive scores
Segment 1	*r* = 0.527 (*p* = .032, corrected *p* = .211)
Segment 2	*r* = 0.559 (*p* = .023, corrected *p* = .141)
Segment 3	*r* = 0.491 (*p* = .042, corrected *p* = .282)
Segment 4	*r* = 0.494 (*p* = .054, corrected *p* = .351)
Segment 5	*r* = 0.451 (*p* = .073, **corrected *p* = .049**)
Segment 6	*r* = 0.323 (*p* = .03, corrected *p* = .213)
Segment 7	*r* = −0.098 (*p* = .71, corrected *p* = 1)
	**CAG repeat length**
Segment 1	*r* = 0.641 (*p* = .002, **corrected *p* = .014**), partial correlation: *r* = 0.763 (*p* = .001, **corrected *p* = .007**)
Segment 2	*r* = 0.717 (*p* = .001, **corrected *p* = .007**), partial correlation: *r* = 0.879 (*p* < .001, **corrected *p* < .001**)
Segment 3	*r* = 0.549 (*p* = .012, corrected *p* = .084), partial correlation: *r* = 0.841 (*p* < .001, **corrected *p* < .001**)
Segment 4	*r* = 0.71 (*p* = .001, **corrected *p* = .007**), partial correlation: *r* = 0.831 (*p* < .001, **corrected *p* < .001**)
Segment 5	*r* = 0.525 (*p* = .018, corrected *p* = .126), partial correlation: *r* = 0.745 (*p* = .001, **corrected *p* = .007**)
Segment 6	*r* = 0.513 (*p* = .021, corrected *p* = .147), partial correlation: *r* = 0.864 (*p* < .001, **corrected *p* < 0.001**)
Segment 7	*r* = 0.107 (*p* = .663, corrected *p* = 1), partial correlation: *r* = 0.5 (*p* = .048, corrected *p* = .336)
	**DBS**
Segment 1	*r* = −0.04 (*p* = .853, corrected *p* = 1)
Segment 2	*r* = 0.08 (*p* = .697, corrected *p* = 1)
Segment 3	*r* = 0.003 (*p* = .986, corrected *p* = 1)
Segment 4	*r* = 0.071 (*p* = .739, corrected *p* = 1)
Segment 5	*r* = 0.048 (*p* = .824, corrected *p* = 1)
Segment 6	*r* = −0.12 (*p* = .642, corrected *p* = 1)
Segment 7	*r* = −0.09 (*p* = .662, corrected *p* = 1)

*Note*: Correlation coefficients that were significant after Bonferroni correction are highlighted in bold. Trends, defined as correlations significant at the uncorrected level, are highlighted in italics.

### 6.5 Whole‐brain analysis with TBCA reveals WM microstructure alterations in the posterior CC, the left CST and the right fronto‐striatal projections

**FIGURE 7:**
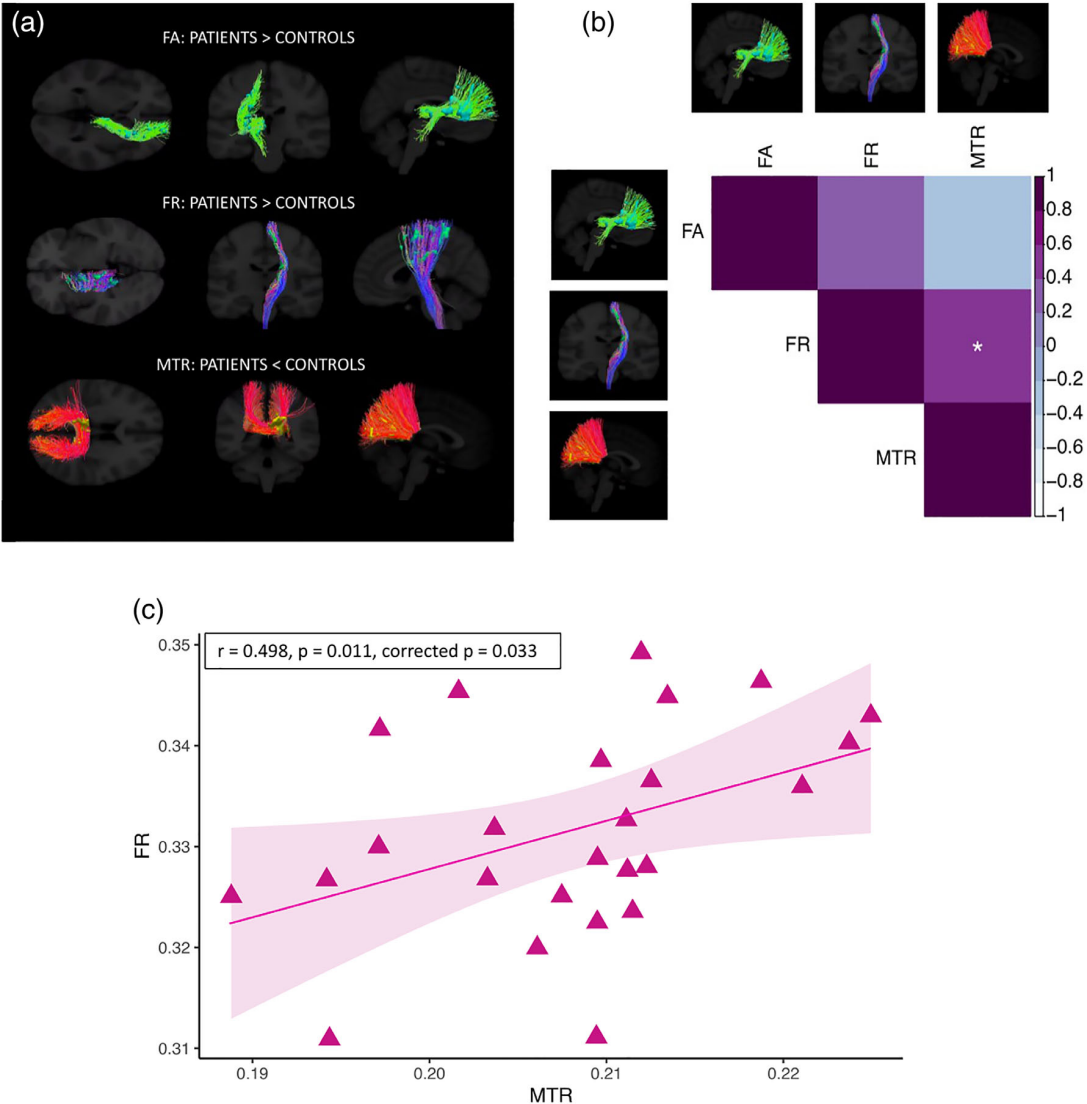
Results of the cluster‐analysis obtained with TBCA between patients and controls (a), Spearman correlations between significant TBCA clusters in patients (b) (*p < .05, **p <0.01, ***p < .001, Bonferroni‐corrected), and plot of MTR in the posterior callosum versus FR in the CST in patients (c)

Figure 7a shows the TBCA results. Consistent with the PCA results, a significant reduction in MTR in the patient group was detected, compared to controls, in the posterior portion of the CC (cluster mass [∑t‐score] = 1,530, *p* < .001 [uncorrected], *p* = .030 [FWE‐corrected]). Furthermore, a significant increase in FR along most of the left CST was found in patients (cluster mass [∑ t‐score] = 1,004, *p* < .001 [uncorrected], *p* = .030 [FWE‐corrected]). Finally, right‐lateralized clusters of significantly higher FA in the patient group were identified in the fronto‐striatal projections (cluster mass [∑t‐score] = 956, *p* < .001 [uncorrected], *p* = .03 [FWE‐corrected]).

Figure 7b plots the relationship between significant microstructure clusters as detected with TBCA for patients. FR in the CST was significantly associated with MTR in the posterior CC (*r* = 0.498, *p* = .011, corrected *p* = .033) (a scatterplot of the relationship is shown in Figure 7c), but not with FA in the right fronto‐striatal projections (*r* = 0.328, *p* = .110, corrected *p* = 0.327). Additionally, MTR was not associated with FA (*r* = −0.218, *p* = .294, corrected *p* = .882).

## 7 DISCUSSION

We carried out a comprehensive tractometry analysis (Bells et al., 2011; Jones et al., 2005; Jones et al., 2006) of regional differences across the CC in premanifest HD compared to age‐ and sex‐matched healthy controls. By exploiting the ultra‐strong magnetic field gradients of the Connectom scanner (Jones et al., 2018; Setsompop et al., 2013), it was possible to better tease apart alterations in myelin/iron content from alterations in axon microstructure (Kleban et al., 2020). Specifically, although measurements of this *style* could be carried out on any scanner, the Connectom allows the realization of high *b*‐values (e.g., *b* = 6,000 s/mm^2^ as used here) with echo times, gradient duration, and gradient separation that cannot be achieved with conventional gradients. Such timing parameters allow diffusion‐weighted data to be acquired with an SNR per unit time that cannot be attained on other MR scanners at present.

We detected lower MTR, but not axon density, in the callosal isthmus of patients compared to controls. These results are consistent with previous DTI studies reporting microstructural changes in this callosal region in premanifest HD (Di Paola, Phillips, Sanchez‐Castaneda, et al., 2014; Phillips et al., 2013). Interestingly, patients presented significantly higher MTR than controls in the callosal rostrum and, overall, MTR was higher in patients than controls in the anterior portions of the CC. Additionally, a positive association was detected between MTR and CAG size, but not DBS, in patients, suggesting a direct link between microstructural alterations and the disease mutation. Finally, a significant interaction effect was detected between group and age on MTR, suggesting that while MTR in this tract is higher in younger patients, the opposite is true for older patients, which likely present increased disease burden.

Our findings may be due to a number of different mechanisms. Based on the high correlations reported between magnetization transfer‐based measures and histological myelin content (Mancini et al., 2020), our results may suggest that, at least early on in disease progression, the HD mutation is associated with excessive, rather than reduced, myelin production. This might be caused by a pathological increase in myelin‐producing oligodendrocytes. In accord with this proposal, previous evidence has suggested that HD gene expression may influence brain cell densities early in the life of gene carriers (Myers et al., 1991), and that increased CAG repeats are associated with more complex neuronal development, including myelination, across species and ontologically (van der Plas et al., 2019). Additionally, this explanation agrees with findings from neuropathology showing increased density of myelin‐producing oligodendrocytes in the brain of premanifest patients (Gómez‐Tortosa et al., 2001). Furthermore *mHTT* directly alters the proliferation property of cultured oligodendrocyte precursor cells (OPCs), with the degree of cell proliferation of OPCs increasing with pathological severity and increasing CAG repeat length (Jin et al., 2015).

As oligodendrocytes are the major iron‐containing cells in the adult central nervous system (Connor & Menzies, 1995), the above studies also support the notion that changes in MTR observed in this study may be driven by iron alterations, and such a proposition is consistent with evidence that changes in iron affect magnetization transfer parameters (Birkl et al., 2020). Crucially, however, as iron and myelin levels in the brain are tightly related, these two explanations are not mutually exclusive, and further work is needed to uncover the generative mechanism underpinning the present findings. Importantly, in accord with these results, recent evidence from the cross‐sectional HD Young Adult Study demonstrated increased R_1_ and R_2_* values, again suggestive of either increased iron or increased myelin, in the putamen, globus pallidum and external capsule of HD patients more than 20 years away from clinical onset (Johnson et al., 2021).

Mutation‐related excessive levels of myelin and/or iron early in the disease may come at the cost of detrimental effects later in the disease due to oxidative stress (Bartzokis et al., 2007; Bartzokis, Cummings, Perlman, Hance, & Mintz, 1999; Bartzokis & Tishler, 2000). Critically, lower MTR in the most posterior callosal areas, through which fibers from the visual system transverse, suggests that these regions are the first to be affected, in agreement with previous evidence (Bartzokis et al., 2007; Coppen, van der Grond, Hafkemeijer, Rombouts, & Roos, 2016; Tabrizi et al., 2009). The visual system is functionally critical early in life, with myelination occurring early and progressing rapidly (Yakovlev, 1967). Additionally, this system is highly dynamic and is associated with big energetic demands. As metabolic dysfunction and alterations in energetics play important mechanistic roles in HD (Beal, 2005; Browne, 2008), these changes may contribute to early microstructural impairment in this callosal portion. The suggestion for myelin impairment in this callosal segment is consistent with a previous study carried out by our group at 7 Tesla (Casella et al., 2021), which demonstrated significantly lower myelin water signal fraction in the posterior callosum of premanifest HD patients. Moreover, this suggestion is in accord with the demyelination hypothesis, which argues that early myelinated fibers are more susceptible to myelin disorder in the disease (Bartzokis et al., 2007).

Overall, we demonstrate measurable and significant differences in callosal magnetization transfer before changes in proxy metrics of axon density can be detected. These changes may reflect early neuronal dysfunction (Rosas et al., 2010) or a CAG‐driven neurodevelopmental component to the pathogenesis of HD, as a precursor to the more global neurodegeneration process (Barnat et al., 2020; Jin et al., 2015; Nopoulos et al., 2010; Phillips et al., 2014). Accordingly, there is increasing evidence that neurodevelopment is affected in HD (Barnat et al., 2020; van der Plas et al., 2019) and that such developmental elements of HD are independent of ongoing neurodegeneration (Johnson et al., 2021). While the present study was not designed to detect HD‐associated developmental changes, future studies following young premanifest subjects longitudinally should address the possibility of toxic myelin levels due to pathological CAG repeats size.

The lack of a significant association between MTR changes and DBS in our study contrasts with previous HD research reporting significant relationships between MRI‐derived measures and cumulative probability to onset (CPO) (Langbehn, Brinkman, Falush, Paulsen, & Hayden, 2004), a measure similar to DBS. Zhang et al.(Zhang et al., 2018), for example, demonstrated that CPO correlated with the neurite density index (NDI) in the callosal body and splenium of HD patients. While MTR and NDI are known to be sensitive to different subcompartments of tissue (i.e., MTR being more sensitive to myelin and NDI being more sensitive to axon density), it is nevertheless useful to speculate as to why one study found a disease burden versus imaging correlation and one did not. First, and foremost, the difference in results may simply reflect heterogeneity of the disease, and the cohorts in the two studies may genuinely have had different underlying microstructural signatures. Second, it may reflect differences in the approaches used to model both microstructure and disease progression in each study. Third, differences in sample sizes across studies (e.g., *n* = 25 in our study compared to, for example, *n* = 38 in Zhang et al. (2018) might have an effect on the observed results. Therefore, it is challenging to assert whether any of the above factors, or indeed their combination, underpinned the difference in findings. Importantly, it has to be noted that a study recently published by Johnson et al. (2021) also reported the lack of significant associations between DBS and any imaging measures assessed, which included, among others, diffusion metrics and proxy measures of myelin and iron content. In their study, Johnson et al. highlight that CSF neurofilament light might be a more sensitive and dynamic marker of the disease course, particularly in premanifest HD (Byrne et al., 2018; Johnson et al., 2021).

With TBCA, clusters of significantly higher FA were detected in the patient group in the right fronto‐striatal projections. Though neurodegenerative disorders have normally been associated with lower FA in major WM pathways, attributed to WM degeneration, demyelination, reduced gliosis or axonal damage as a result of GM loss (Assaf, 2008; Concha, Gross, Wheatley, & Beaulieu, 2006), it is possible that selective degeneration of specific WM tracts resulted here in higher anisotropy values and a paradoxical increase in microstructural organization (Douaud et al., 2009). This suggests that WM degeneration in this area is already present at the premanifest stage of the disease.

Importantly, significantly higher FR along most of the left CST was also detected with TBCA. This tract is composed of descending WM fibers, with half of them arising from the primary motor cortex, and is anatomically linked to the basal ganglia (Kandel, Schwartz, & Jessell, 2000; Schultz, 2001). From a functional point of view, the CST conducts motor impulses from the brain to the spinal cord, and plays an essential role in voluntary movement (Kandel et al., 2000; Schultz, 2001). Though the hallmark symptom of HD concerns involuntary choreic movements (Folstein, 1989), alterations in voluntary movement are also present in premanifest patients (Rowe et al., 2010), thus suggesting that alterations in this tract may play an important role in the disease. Crucially, this is the first time that alterations in this measure have been detected in premanifest patients, pointing to the potential of FR as in‐vivo MRI marker of premanifest neural changes.

Previous studies have demonstrated lower WM volume in the internal capsule of manifest patients (Fennema‐Notestine, Archibald, Jacobson, et al., 2004; Nave, Ginestroni, Tessa, et al., 2010). Accordingly, the elevated FR detected in this study might reflect the loss of non‐neuronal cells, in turn leading to axons being pushed together (Rattray et al., 2013). Alternatively, such a result might reflect axonal swelling (Marangoni et al., 2014). Consistent with this suggestion, previous evidence demonstrated higher iron levels in the left CST of premanifest patients (Johnson et al., 2021; Phillips et al., 2015), interpreted as indicating an homeostatic increase in oligodendrocytes to repair myelin damage. In turn, myelin damage leads to axon swelling (Payne, Bartlett, Harvey, Dunlop, & Fitzgerald, 2012). It might also be that fiber bundles develop differently because of the genetic mutation, and this is consistent with evidence of morphological alterations in the neurons of HD mice, which present smaller diameter dendritic shafts, smaller somatic cross‐sectional areas, and decreased diameter of the dendritic fields (Klapstein et al., 2001). Finally, higher FR might reflect the presence of a process of reorganization and compensatory pruning of axons in WM, such as pathologically driven reduced collateral branching or morphological alterations of individual axons. Consistent with this suggestion, evidence has shown increased coherence of axonal organization in premanifest patients, as suggested by a smaller orientation dispersion index (OD), in tracts surrounding the basal ganglia and in the internal and external capsule (Zhang et al., 2018). Additionally, the significant association between FR in the CST and MTR in the posterior callosum, further suggests the presence of compensatory mechanisms involving the WM of patients.

Finally, the finding of higher FR in the left CST is consistent with the leftward‐biased GM loss demonstrated in the striatum of patients (Muhlau, Gaser, & Wohlschager, 2007) and with the leftward asymmetry of brain iron in aging and motor disorders (Langkammer et al., 2010; Xu, Wang, & Zhang, 2008). Nevertheless, future studies are needed to determine whether this is an important finding to understand disease pathology. For example, future studies could investigate the longitudinal evolution of changes in FR in patients.

### 7.1 Study limitations and future directions

To date, only one other study has used extensive microstructural measures in premanifest HD (Johnson et al., 2021). Moving beyond commonly‐available diffusion tensor imaging measures, and using such advanced measurements is essential for understanding the trajectory of WM microstructure alterations across the disease course, which is expected to vary as disease processes change (Johnson et al., 2021; Lommers et al., 2019). Notably, though much of our understanding of HD pathology will increasingly rely on advanced neuroimaging techniques, it is important to remember and address the shortcomings of these approaches. Accordingly, while it is tempting to assign, unequivocally, a one‐to‐one correlation between changes in the MRI signal and biological properties, the present findings need to be interpreted with caution.

For example, it is important to note that the MTR is influenced by a complex combination of biological factors (including T_1_), making it difficult to pinpoint with certainty which pathological processes are responsible for the altered MTR observed in patients in this study. While a change in myelination will result in a change in MTR, a change in MTR may result from other physiological /biophysical changes in the WM (including changes in T_1_), making it difficult to separate the effects of reduced macromolecular density because of demyelination and/or axonal loss, iron alterations or increased water because of oedema and/or inflammation (Deloire‐Grassin, Brochet, Quesson, et al., 2000; Dousset et al., 1992; Dousset et al., 1995; Gareau, Rutt, Karlik, & Mitchell, 2000). Therefore, though an attempt was made to control for confounding elements by, for example, including FWF as a factor in the analyses, and complementing MTR with other microstructure‐sensitive metrics, these results require replication in future studies. More specifically, future investigations may benefit from utilizing quantitative magnetization transfer (Henkelman et al., 1993), myelin water imaging (MacKay & Laule, 2016) or inhomogeneous magnetization transfer (Ercan et al., 2018) to assess myelin alterations in the premanifest disease stage.

A similar methodological consideration needs to be made with regards to the interpretation of FR changes. Specifically, because of the way FR is computed (i.e., the CHARMED model recovers a T2‐weighted restricted and hindered diffusion‐weighted signal), variation in T_2_ relaxation (e.g., because of altered tissue water or myelin content) may be erroneously interpreted as a difference in FR.

Additionally, it is challenging to estimate the contribution of smaller axons to the diffusion signal (Drakesmith et al., 2019). Though this work utilized ultra‐strong gradients (300 mT/m), therefore allowing the contribution of axons with a diameter as small as 3 μm to be assessed (Drobnjak, Zhang, Ianuş, Kaden, & Alexander, 2016; Nilsson, Lasič, Drobnjak, Topgaard, & Westin, 2017; Sepehrband, Alexander, Kurniawan, Reutens, & Yang, 2016), the majority of axons in the brain have a diameter smaller than 1 μm (Aboitiz et al., 1992; Caminiti et al., 2013; Liewald, Miller, Logothetis, Wagner, & Schüz, 2014; Sepehrband et al., 2016). Because of this, changes in later myelinating WM areas (such as the anterior portions of the CC), which are characterized by small and thinly myelinated axons, may have not been appropriately reflected by variation in FR. Hence, there is a possibility that increases in MTR observed in the anterior portions of the CC may have reflected decreased axonal density in this area, rather than compensatory remyelination. However, the lack of significant changes in other measures, such as AD or RD, suggests the absence of significant axon changes in the HD sample.

To gain increased understanding of the neurobiological underpinnings of FR differences, future studies could investigate disease‐associated changes in volume and axon diameter distribution in the CST. Additionally, they might assess apparent fiber density changes at high diffusion‐weightings, to increase suppression of the extra‐axonal signal. This approach was recently shown to enable a better characterization of microstructural changes, because of the improved correspondence with intra‐axonal properties (Genc et al., 2020; Kleban et al., 2020; McKinnon & Jensen, 2019).

Finally, our findings were based on a relatively small sample size and warrant replication in larger samples, which could additionally benefit from being assessed longitudinally rather than cross‐sectionally, to enable a better understanding of how imaging changes relate to clinical symptoms over time, and evaluate the utility of these metrics as markers of early disease development and progression.

Notwithstanding the above limitations, findings from this work highlight the fundamental importance of gaining an enhanced understanding of the mechanisms underlying WM abnormalities in HD. Crucially, our results suggest that microstructure alterations in the disease may reflect CAG‐driven neurodevelopmental, rather than neurodegenerative, changes and that expanding intervention strategies to include oligodendroglial targets (Bardile et al., 2019) directly targeting WM pathology may be beneficial for HD.

## Data Availability

The data analysed during the current study and the respective analysis scripts are available from the corresponding author on reasonable request.
